# Nanocrystallized Ge-Rich SiGe-HfO_2_ Highly Photosensitive in Short-Wave Infrared

**DOI:** 10.3390/ma14227040

**Published:** 2021-11-20

**Authors:** Catalin Palade, Ana-Maria Lepadatu, Adrian Slav, Valentin Serban Teodorescu, Toma Stoica, Magdalena Lidia Ciurea, Doru Ursutiu, Cornel Samoila

**Affiliations:** 1 National Institute of Materials Physics, 405A Atomistilor Street, 077125 Magurele, Romania; catalin.palade@infim.ro (C.P.); slav@infim.ro (A.S.); teoval@infim.ro (V.S.T.); 2 Academy of Romanian Scientists, 54 Splaiul Independentei, 050094 Bucharest, Romania; udoru@unitbv.ro; 3 Department of Electronics and Computer Science, Transylvania University of Brasov, 29 Eroilor, 500036 Brasov, Romania; 4 Department of Materials Science, Transylvania University of Brasov, 29 Eroilor, 500036 Brasov, Romania; csam@unitbv.ro; 5 Romanian Technical Science Academy, 26 Dacia, 010413 Bucharest, Romania

**Keywords:** group IV nanocrystals, HfO_2_, magnetron sputtering, rapid thermal annealing, SWIR, spectral photocurrent

## Abstract

Group IV nanocrystals (NCs), in particular from the Si–Ge system, are of high interest for Si photonics applications. Ge-rich SiGe NCs embedded in nanocrystallized HfO_2_ were obtained by magnetron sputtering deposition followed by rapid thermal annealing at 600 °C for nanostructuring. The complex characterization of morphology and crystalline structure by X-ray diffraction, μ-Raman spectroscopy, and cross-section transmission electron microscopy evidenced the formation of Ge-rich SiGe NCs (3–7 nm diameter) in a matrix of nanocrystallized HfO_2_. For avoiding the fast diffusion of Ge, the layer containing SiGe NCs was cladded by very thin top and bottom pure HfO_2_ layers. Nanocrystallized HfO_2_ with tetragonal/orthorhombic structure was revealed beside the monoclinic phase in both buffer HfO_2_ and SiGe NCs–HfO_2_ layers. In the top part, the film is mainly crystallized in the monoclinic phase. High efficiency of the photocurrent was obtained in a broad spectral range of curves of 600–2000 nm at low temperatures. The high-quality SiGe NC/HfO_2_ matrix interface together with the strain induced in SiGe NCs by nanocrystallization of both HfO_2_ matrix and SiGe nanoparticles explain the unexpectedly extended photoelectric sensitivity in short-wave infrared up to about 2000 nm that is more than the sensitivity limit for Ge, in spite of the increase of bandgap by well-known quantum confinement effect in SiGe NCs.

## 1. Introduction

There is considerable interest in nanostructured materials from the group IV Si–Ge system for photonics applications [1,2,3,4,5,6,7,8,9,10]. However, the most important inconvenience of this system is the low light absorption-emission efficiency of bulk Si–Ge with an indirect band gap that counts against the long-held goal of integrated group IV photonics. This inconvenience can be solved by nanostructuring (quantum confinement) combined with strain [11,12,13] or by exploiting other crystalline structures different from *Fd*-3*m* diamond [14,15], such as metastable hexagonal phase [16]. Other solutions are to develop plasmonic structures [17], to fabricate structures with GeSi quantum dots (QDs) embedded in microresonators [18], or by employing hydrogenation technique for passivating detrimental defects [19], all these enabling the enhancement of GeSi and Ge QDs photoluminescence. Another proposed route targeted the host matrix considering its role in the Ge QD growth kinetics and morphology (QDs density and separation distances) and in the light absorption of Ge QDs embedded in matrix, i.e., better Si_3_N_4_ instead of SiO_2_ [20]. For photodetectors based on nanocrystals (NCs)/QDs from the Si–Ge system, it was shown that the photoresponse is enhanced by NCs formation (and strain) and is size-dependent, the spectral sensitivity limit being controlled by NC size [21,22,23,24]. Additionally, photocurrent enhancement can be further induced by field effects [23,25] or achieved by coating Ge QDs with metal shells [26]. Photoconductive properties can also be boosted, besides quantum confinement effect in Ge NCs/QDs, by exploiting the Ge related defects/traps, most of them being localized states from NC/oxide matrix interface, by trapping photogenerated holes on these defects/traps and thus increasing the lifetime of electrons [27,28,29]. Recently, a phototransistor with Ge QDs decorated on a single Si-nanowire channel integrated on an SOI platform was reported, its spectral response at room temperature ranging from 1200 to 1700 nm [30].

On the other hand, short wave infrared (SWIR) extended spectral photocurrent is ensured by stabilization of Ge-rich alloy SiGe NCs [25] against *fast Ge diffusion* [31] as we showed for Ge-rich SiGe NCs embedded in TiO_2_. This is achieved by preparing NCs surrounded by SiO_2_ thin layers that have a protective role against *Ge fast diffusion*, resulting in the formation of Ge-rich SiGe NCs. Thus, SWIR spectral sensitivity (cut-off wavelength) was pushed up to 1700 nm for cooled structures (100 K), and up to 1400 nm at room temperature [25], in comparison with Ge NCs embedded in TiO_2_ for which a cut-off of 1250 nm was obtained [23]. Density functional theory (DFT) computations of the energy gap of alloy GeSi NCs with high Ge content for finding bandgap diameter dependence for different Ge contents and bigger diameters in agreement to the experimental data can be used for the design and characterization of GeSi NCs-based optical sensors [32]. Based on all these results, recently, we developed an optical sensor system with a photoactive layer of GeSi NCs in the SiO_2_ matrix that discriminates between different slippery road conditions, to be mounted on a platform for warning drivers in due time and at sufficient distance [33]. The sensor has 360–1350 nm sensitivity and 10^2^–10^3^ signal/noise ratio at room temperature. As shown in the present paper, the SWIR photoresponse can be further improved by using nanocrystalline HfO_2_ as the embedding matrix instead of SiO_2_ or TiO_2_. HfO_2_ is a high-k oxide, highly CMOS compatible, and highly scalable. The nanocrystalline HfO_2_ as matrix creates a good separation between SiGe NCs and thus the SiGe NCs/HfO_2_ NCs matrix interface is of high quality [34]. Using HfO_2_ as a matrix is beneficial as it allows the obtaining of SiGe NCs-HfO_2_ films with good NCs surface passivation by HfO_2_ oxide matrix.

In this paper, films of Ge-rich SiGe NCs embedded in nanocrystallized HfO_2_ were prepared by magnetron sputtering deposition of 3-layers stacks of *cap HfO_2_/SiGeHfO_2_ active layer/buffer HfO_2_/on Si* followed by rapid thermal annealing (RTA). The structures present a cut-off wavelength extended in SWIR of about 2000 nm in the broad 600–2000 nm spectral photocurrent of the structures at low temperatures, due to photocarrier generation in the heterojunction of the embedded Ge-rich SiGe NCs with 0.6% evaluated strain and Si substrate. The cap and buffer are very thin HfO_2_ layers hampering the *fast diffusion of Ge* from the active layer, without blocking the electrical contact to the substrate and top contacts.

## 2. Materials and Methods

### 2.1. Sample Preparation

Photosensitive films of Ge-rich SiGe NCs embedded in nanocrystallized HfO_2_ are prepared by magnetron sputtering deposition (Surrey Nanosystems Gamma 1000 equipment, Surrey NanoSystems Ltd., Newhaven, United Kingdom) of 3-layers stack of *cap HfO_2_/SiGeHfO_2_ active layer/buffer HfO_2_/Si wafer* followed by RTA (Annealsys AS-Micro rapid thermal prcessing system, Annealsys, Montpellier, France) for nanostructuring. The (*Si_1–x_Ge_x_)_1–y_(HfO_2_)_y_ layer* is obtained by co-deposition from 3 independent plasma controllers of targets of Si (15 W DC power), Ge (10 W DC), and HfO_2_ (45 W RF), the estimated volume composition (based on deposition rates) being *x* = 70% and *y* = 50%. *Buffer* and *cap HfO_2_* layers that are cladding the *active SiGe-based layer* are deposited by sputtering from the HfO_2_ target. Substrates of cleaned p-*Si* with 7–14 Ωcm resistivity were used, and sputtering was made in Ar atmosphere at 4 mTorr. *Buffer HfO_2_* and *cap HfO_2_* layers serve to reduce the Ge loss by *fast diffusion* from the photoactive SiGe region. After nanocrystallization by RTA at 600 °C for 8 min in Ar (6N purity) atmosphere, SiGe NCs and HfO_2_ NCs are formed. The resulting structure has layer thicknesses of 160 nm for the *SiGe NCs–HfO_2_* photosensitive layer, and 17 and 9 nm for *buffer* and *cap HfO_2_* layers, respectively. For photoelectrical measurements, Al coplanar contacts with 5 × 2 mm^2^ areas and 2 mm gap between them were deposited by vacuum evaporation on top of annealed structures.

### 2.2. Measurement Methods

Structure, morphology, and composition characterization was made by cross-section transmission electron microscopy (XTEM—Jeol JEM-ARM 200F electron microscope, JEOL Ltd., Tokyo, Japan), X-ray diffraction (XRD—Rigaku SmartLab, Rigaku, Tokyo, Japan), and Raman spectroscopy (Horiba LabRAM HR Evolution μ-Raman spectrometer with laser excitation wavelengths of 633 and 325 nm, HORIBA France SAS, Loos, France).

Spectral photoresponsivity was measured by using the lock-in amplifier technique, under illumination with monochromatic light modulated at 120 Hz, in a dedicated set-up (more details in [25]). Second-order blocking was ensured by longwave pass filters (550, 1000, 1150, and 1250 nm cut-on wavelengths). Measurements were performed in both photovoltaic regimes (0 V voltage) and by applying 1 V bias voltage.

## 3. Results and Discussion

### 3.1. XTEM Analysis

**Figure 1:**
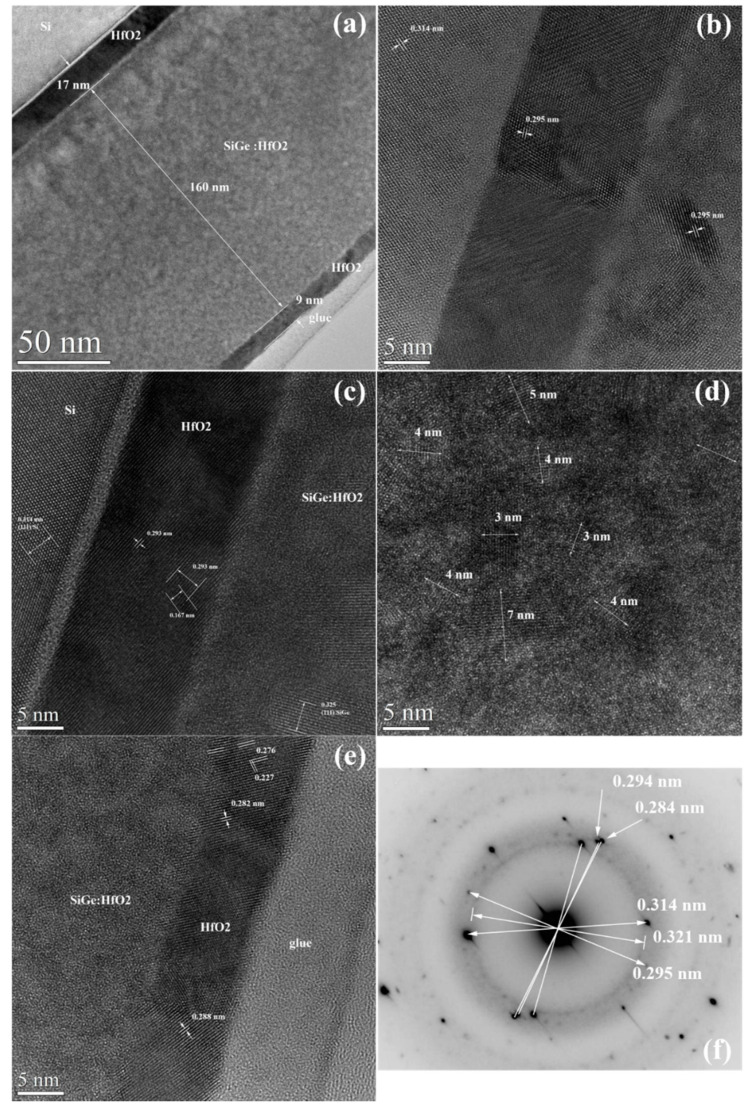
XTEM analysis: (**a**) general view of *9 nm* *HfO_2_/160 nm SiGe NCs–HfO_2_/17 nm HfO_2_/2 nm SiO_2_/Si* structure; (**b**,**c**) HRTEM images in the bottom part of annealed structure near the interface with the Si substrate; (**d**) HRTEM image from *SiGe NCs–HfO_2_* layer; (**e**) HRTEM image in the *top* part near the free surface of annealed structure; (**f**) SAED pattern of *HfO_2_/SiGe NCs–HfO_2_/HfO_2_/SiO_2_/ Si* ((111) 0.314 nm spots) structure.

The general cross-section view of samples is given in Figure 1a which reveals the 160 nm thick *SiGe NCs–HfO_2_* photosensitive layer cladded by *buffer* and *cap* *HfO_2_* layers. The *HfO_2_/SiGe NCs–HfO_2_/HfO_2_/SiO_2_/on Si* structure has a thickness of 9 nm/160 nm/17 nm/2 nm with an error of ±1 nm. HRTEM images in the bottom part of the annealed structure near the interface with the Si substrate (Figure 1b,c) show the nanocrystallization of HfO_2_ with tetragonal/orthorhombic structure (0.293–0.295 nm lattice fringes) in both *buffer HfO_2_* and *SiGe NCs–HfO_2_* layers. Figure 1c evidences a SiGe NC by the (111) 0.325 nm lattice fringes corresponding to a high Ge atomic composition of 87%. HRTEM image in Figure 1d shows that the *SiGe NCs–HfO_2_* layer is formed of both SiGe and HfO_2_ NCs with sizes from 3 to 7 nm, the average size being 4 nm. The *top HfO_2_* layer has a monoclinic structure (Figure 1e). SAED pattern from Figure 1f reveals the cubic structure of SiGe NCs by 0.321 ± 0.002 nm (111) reflections. The presence of tetragonal HfO_2_ is also evidenced by the 0.294 (111) reflections [34], while monoclinic HfO_2_ structure is shown by 0.284 (111) reflections [35]. If we consider the SAED value of 0.321 ± 0.002 nm, then the Ge concentration of SiGe NCs is 57% ± 13%, close to the atomic Ge concentration of about 50% estimated based on deposition rate calibration.

### 3.2. XRD Investigations

**Figure 2:**
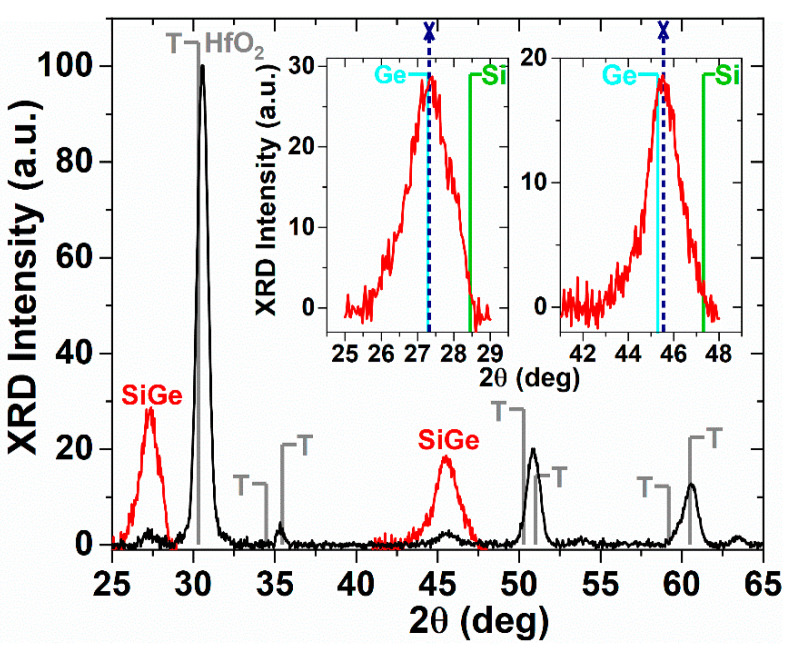
XRD diffractograms of annealed structures: full diffraction curve from 25 to 65 deg (black) and long-time scans around the SiGe 111 and 220 lines of the cubic structure (insets)—red color. The 111 and 220 maxima positions corresponding to Ge-rich SiGe NCs are indicated by navy short dot lines ended with X symbol. The positions of 111 and 220 lines of pure Si (PDF 01-070-5680) in green and Ge (PDF 004-0545) in cyan are shown. Tetragonal HfO_2_ most intense lines labeled T according to PDF 01-078-5756 are also indicated (gray).

The diffractogram measured on the annealed sample is given in Figure 2. Most intense maxima correspond to different crystallographic planes of the tetragonal T HfO_2_ phase. Monoclinic HfO_2_ was not detected (according to PDF 01-078-0049, most intense lines are at 28.35 and 31.65 deg). This is in good agreement with HRTEM results, i.e., nanocrystallization of HfO_2_ with T structure (0.293–0.295 nm lattice fringes) evidenced along with a great thickness of structure (~177 nm) in both *buffer HfO_2_* (17 nm thickness) and *SiGe NCs–HfO_2_* layer (160 nm), while the thin *top HfO_2_ cap* layer (9 nm) has monoclinic structure. This means that the tetragonal T component is dominant in the structure, in good agreement with the global XRD curve.

However, two maxima with low intensity corresponding to SiGe 111 and 220 lines of the cubic structure are measured. These SiGe maxima corresponding to SiGe NCs are better evidenced by long-time scans around the SiGe 111 and 220 lines (Figure 2—insets) [36]. Ge concentration *x* of Si_1−x_Ge_x_ NCs evaluated from the maxima positions by linear interpolation between positions corresponding to pure Si and Ge is 95% Ge for the (111) peak and 88% for the (220) peak. The Ge atomic concentration in SiGe NCs found by XRD and HRTEM analyses, higher than the mean value in the active *SiGeHfO_2_* layer, can be explained by a *fast diffusion of Ge* than Si to form SiGe NCs. However, the strain induced by HfO_2_ nanocrystallization can influence the estimation of composition in SiGe NCs. The average size of SiGe NCs calculated by using the full width at half-maximum of the (111) and (220) peaks is 6.1 and 4.9 nm, respectively in good agreement with the HRTEM results (3–7 nm).

### 3.3. Raman Scattering Analysis

**Figure 3:**
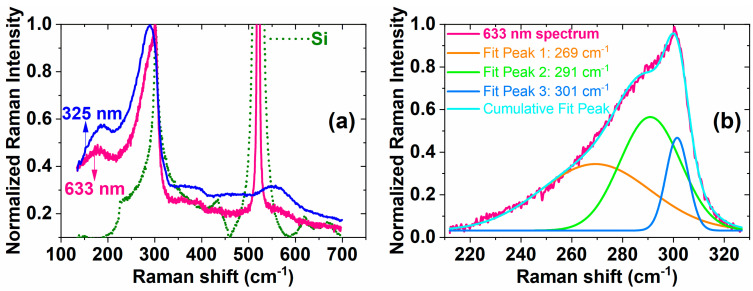
Raman scattering results: (**a**) Raman spectra measured on RTA sample with laser excitation wavelengths of 633 and 325 nm compared with Raman spectrum measured on bulk Si (633 nm excitation), sample spectra are normalized to the intensity of Ge–Ge peak, while the Si spectrum is normalized to the intensity of peak corresponding to the 2TA mode of Si wafer (~301 cm^−1^); (**b**) normalized Raman spectrum of sample after subtracted straight line procedure in the 210–330 cm^−1^ range and deconvolution of Gauss peaks with corresponding maximum position at 269, 291 and 301 cm^−1^ together with cumulative peak fit curve.

Raman spectra measured on the RTA sample with laser excitation wavelengths of 633 and 325 nm are presented in Figure 3a and are compared with the Raman spectrum measured on bulk Si (633 nm excitation). The sample spectra are normalized to the intensity of the Ge–Ge peak, while the Si spectrum is normalized to the intensity of peak corresponding to the 2TA mode of the Si wafer (~301 cm^−1^). The 633 nm spectrum presents the 2TA mode of Si substrate and a shoulder positioned around 291 cm^−1^ together with a broad band (centre at ~269 cm^−1^) as evidenced by the deconvolution from Figure 3b. The maximum positioned at ~291 cm^−1^ corresponds to Ge–Ge vibration mode in Ge-rich SiGe NCs, being shifted to lower energy due to the quantum confinement and stress effect. We evaluated the strain in SiGe NCs as being 0.6% [2] by considering a Ge content of 70% in the SiGe NCs.

The broad band centred at ~269 cm^−1^ (Figure 3b) is the signature of the SiGe disordered component [25]. The 325 nm spectrum reveals only the contribution of Ge-rich SiGe NCs in nanocrystallized HfO_2_ matrix due to stronger absorption in the SiGe-containing layer (Electronic Supplementary Information of Ref. [37]).

### 3.4. Spectral Photosensitivity

**Figure 4:**
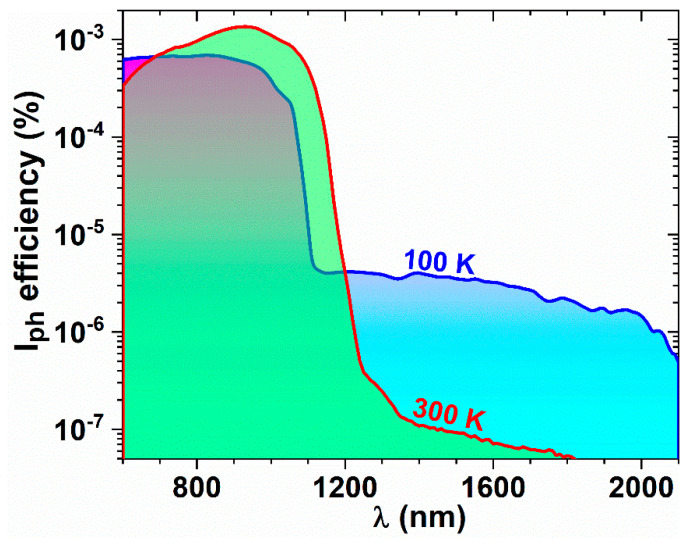
Spectral photocurrent efficiency measured at 100 and 300 K in photovoltaic regime.

The spectral photocurrent efficiency measured in the photovoltaic (0 V) regime at 100 and 300 K is illustrated in Figure 4. One can see that at 100 K the samples are sensitive in visible—near-infrared—short wave infrared (VIS–NIR–SWIR) from 600 nm up to about 2000 nm. The photoresponsivity at 100 K extends in SWIR at longer wavelengths than expected for bulk Ge-rich SiGe. This can be explained by strain induced in SiGe NCs that drastically reduces the bandgap, but also by crystalline disorder in SiGe NCs that results in extension of the electronic states distribution into the bandgap. We have to mention that the spectral photocurrent efficiency is slightly increased by only 17% by applying a voltage bias of 1 V in comparison to 0 V bias. As can be seen in Figure 4, the photocurrent spectrum is also shifted to longer wavelengths by increasing the measurement temperature at 300 K, but the efficiency is strongly reduced due to the increase of the photocarrier recombination probability. We showed in previous reports on group IV-based NCs that the spectral photocurrent efficiency is much increased at low temperatures (100–200 K) than at room temperature, and it has a weak dependence on temperature in contrast to the 200–300 K interval for which it strongly decreases with the increase of temperature (e.g., in [38]). The lowering of the efficiency at high temperature reduces the photocurrent below the sensitivity of the used measurement setup for wavelengths longer than 1800 nm. The SWIR photosensitivity is due to the light absorption in the heterojunction region of Ge-rich SiGe NCs separated by a very thin *HfO_2_ buffer* layer from the Si substrate [25,39]. The high sensitivity detected in our samples is due to the high-quality interface of SiGe NCs with the nanocrystallized HfO_2_ matrix. The beneficial role of using nanocrystallized HfO_2_ matrix we also proved in *gate HfO_2_/floating gate of Ge QDs in HfO_2_/tunnel HfO_2_* memory capacitors with high memory performance by obtaining floating gates consisting of a single layer of well-separated Ge QDs in nanocrystallized HfO_2_, Ge QDs being located at the crossing of the HfO_2_ NCs boundaries [34].

The broad spectral photosensitivity of the investigated photosensor based on Ge-rich SiGe NCs can have multiple applications such as monitoring of slippery road conditions, internet of things, and biomedical applications.

## 4. Conclusions

We have shown that by embedding Ge-rich SiGe NCs in nanocrystallized HfO_2_, the 3-layers stack of *cap HfO_2_/SiGe NCs in HfO_2_ active layer/buffer HfO_2_/Si wafer* has enhanced sensitivity in SWIR. Specific to this 3-layers stack is the nanocrystallized HfO_2_ with tetragonal/orthorhombic structure that was revealed beside the monoclinic phase in both *buffer HfO_2_* and *SiGe NCs–HfO_2_* layers. The high-efficiency photocurrent with broad 600–2000 nm spectral response in VIS–NIR–SWIR measured at 100 K shows extended cut-off wavelength to 2000 nm. This is explained by the high quality of the SiGe NC/HfO_2_ interface together with the strain induced in Ge-rich SiGe NCs (3–7 nm diameter and 0.6% evaluated strain) that drastically reduces the bandgap.

## Data Availability

The data presented in this study are available on request from the corresponding authors.
